# Effectiveness of home- and community-based services in decreasing health care service and expenditure in Taiwan

**DOI:** 10.1093/geroni/igab046.3591

**Published:** 2021-12-17

**Authors:** Ya-Mei Chen, Shih-Cyuan Wu, Shiau-Fang Chao, Kuan-Ming Chen, Chen-Wei Hsiang, Ming-Jen Lin, Ji-Lung Hsieh, Kuan-Ju Tseng

**Affiliations:** 1 National Taiwan University, Taipei, Taipei, Taiwan (Republic of China); 2 National Taiwan University, Taipei City, Taiwan (Republic of China); 3 National Bureau of Economic Research, CAMBRIDGE, Massachusetts, United States; 4 National Taiwan University, National Taiwan University, Taipei, Taiwan (Republic of China); 5 Graduate Institute of Journalism, Taipei City, Taipei, Taiwan (Republic of China)

## Abstract

Background Whether long-term care service use decreases older adults’ health care service use and cost has been a strong interest among aging countries, including Taiwan. The current study examined the impact of continuous use of HCBS offered by Taiwan’s LTC plan 2.0 on older adults’ health service utilization and cost overtime. Methods This study used the LTC Plan 2.0 database and the National Health Insurance Plan claim dataset, and included 151,548 clients who had applied for and were evaluated for LTC services for the first time from 2017 through 2019 and continuously used any LTC Plan 2.0 services for six months. Outcome variables were users’ health service utilization and health care cost 12 months before and after starting to continuously use HCBS. Latent class analysis and generalized estimating equations were used to investigate the influences of different service use patterns on the changes in physical functions. Results Three subgroups of LTC recipients with different use patterns, including home-based personal care (home-based PC) services (n = 107324, 70.8%), professional care services (n = 30466, 20.1%), and community care services (n = 13794, 9.1%) were identified. When compared to care recipients in the community care group, those in the home-based PC group had more emergency room expenditures (1 point/month, p< 0.05) but less hospitalization expenditures (38 points/month, p<0.001), while the professional care group had less emergency room and hospitalization expenditures (3 and 138 points/month, p< 0.001). Conclusion Those receiving professional care and home care services spent less on health care service utilization.

